# Lumbar enlargement spinal cord stimulation for severe spasticity and motor function improvement after traumatic brain injury: a case report

**DOI:** 10.3389/fnhum.2026.1754152

**Published:** 2026-03-11

**Authors:** Di Wu, Yaping Wang, Bo Hong

**Affiliations:** 1 Department of Pain Management, The Second Xiangya Hospital, Central South University, Changsha, Hunan, China; 2 Department of Anesthesiology, The Second Xiangya Hospital, Central South University, Changsha, Hunan, China; 3 Yueyang Traditional Chinese Medicine Hospital, Yueyang, Hunan, China

**Keywords:** case report, refractory, spasticity, spinal cord stimulation, traumatic brain injury

## Abstract

This case report describes the successful use of epidural spinal cord stimulation (SCS) in managing severe, refractory spasticity in a 58-year-old male following traumatic brain injury. Despite nearly 8 months of conventional pharmacotherapy and rehabilitation for his tetraplegia, his lower-limb spasticity persisted at Modified Ashworth Scale (MAS) grade 3, severely impeding functional recovery. After implantation of a trial and subsequently permanent SCS system at the lumbar enlargement, muscle tone decreased to MAS grade 2 within 48 h, alongside improvements in muscle strength. Over 6 months, stimulation led to a marked reduction in the frequency and severity of spastic episodes. This spasticity relief fundamentally improved the patient’s sleep quality and enabled significant functional gains, including assisted standing and pedal stepping. This case demonstrates the positive effect of SCS for a condition often resistant to standard treatments. The results support re-evaluating SCS’s therapeutic potential for refractory spasticity caused by TBI and other central nervous system disorders, potentially through mechanisms involving the modulation of spinal cord excitability.

## Introduction

Traumatic brain injury (TBI) results from external forces acting on the head, which can cause severe and permanent brain damage (Dobson et al., 2024). It is one of the leading causes of disability worldwide, affecting over 50 million people annually and imposing an economic burden exceeding 400 billion US dollars on the global economy. Besides cognitive and sensory impairments, spasticity is a common secondary motor dysfunction following TBI, with an incidence ranging from 30 to 50% (Synnot et al., 2017).

Spasticity is clinically recognized as a disorder of sensorimotor control, characterized by a velocity-dependent increase in stretch reflexes, along with hyperactive tendon reflexes, and is considered a component of Upper Motor Neuron Syndrome (UMNS) (Tamburin et al., 2022). In individuals with TBI, spasticity may manifest as early as 1 week following the injury and tends to exacerbate as the condition evolves. Its characteristic manifestations include abnormal muscle hypertonia, involuntary oscillatory contractions of the affected muscles, increased amplitude of stretch reflexes (hyperreflexia), and pathological co-contraction patterns of agonists and antagonists (Bose et al., 2015). This condition of patients with TBI predominantly affects the antigravity muscle groups; in the lower extremities, it primarily involves the hip adductors, knee flexors, and ankle plantar flexors and invertors, while in the upper extremities, it chiefly impacts the shoulder adductors and the flexors of the elbow, wrist, and fingers. The musculoskeletal complications associated with spasticity, such as muscle contractures, involuntary tremors, joint stiffness, diminished range of motion, and pain, can significantly hinder the patient’s capacity to participate in social activities and may ultimately result in disability (Subramanian et al., 2022).

The pathophysiological essence of spasticity primarily originates from the imbalance between supraspinal inhibitory control and excitatory drive. After the disease or injury that causes spasticity, excitatory signals dominate and override the control of inhibitory signals. Its pathophysiological changes can be divided into two categories: spinal mechanisms involving motor neurons and interneurons, and supraspinal and suprasegmental mechanisms. The imbalance of reflex circuits and neuronal plasticity in the spinal cord, as well as the imbalance of descending pathways above the spinal cord, jointly mediate the clinical manifestations of spasticity (Raghavan, 2025; Mukherjee and Chakravarty, 2010).

Current clinical management of spasticity mainly includes pharmacological and non-pharmacological treatments. Pharmacological treatments include oral medications (such as baclofen, tizanidine, dantrolene) and local injection therapies using local anesthetics or botulinum toxin A. Non-pharmacological treatments include various invasive procedures such as intrathecal baclofen pump (ITB), selective dorsal rhizotomy (SDR), and spinal cord stimulation (SCS). However, oral medications have systemic side effects (sedation, muscle weakness) and limited efficacy; botulinum toxin requires repeated injections; ITB has difficulties in drug titration, risk of drug tolerance, catheter-related complications, and overdose (Brandenburg et al., 2023). SCS was initially used for pain treatment and has been applied to movement disorders since the 1970s. In the 1970s–1980s, more than 1,000 patients received stimulator implants targeting motor circuits. Since the 21st century, especially in recent years, new evidence has emerged regarding the efficacy of SCS in treating spasticity. SCS directly activates spinal inhibitory neural circuits and regulates the excitability of motor neurons, and the therapeutic potential of epidural spinal cord stimulation in spasticity management deserves attention (Nagel et al., 2017).

This article reports a case of a patient with severe lower limb spasticity after severe TBI. After standard pharmacological and rehabilitation treatments failed, the patient received spinal cord stimulation (SCS) implantation in the Lumbar Enlargement region and achieved remarkable efficacy. By detailing the diagnosis and treatment process, assessment methods, and mechanism exploration, this case provides clinical evidence for the application of SCS in spasticity management.

### Case study presentation

**Figure 1:**
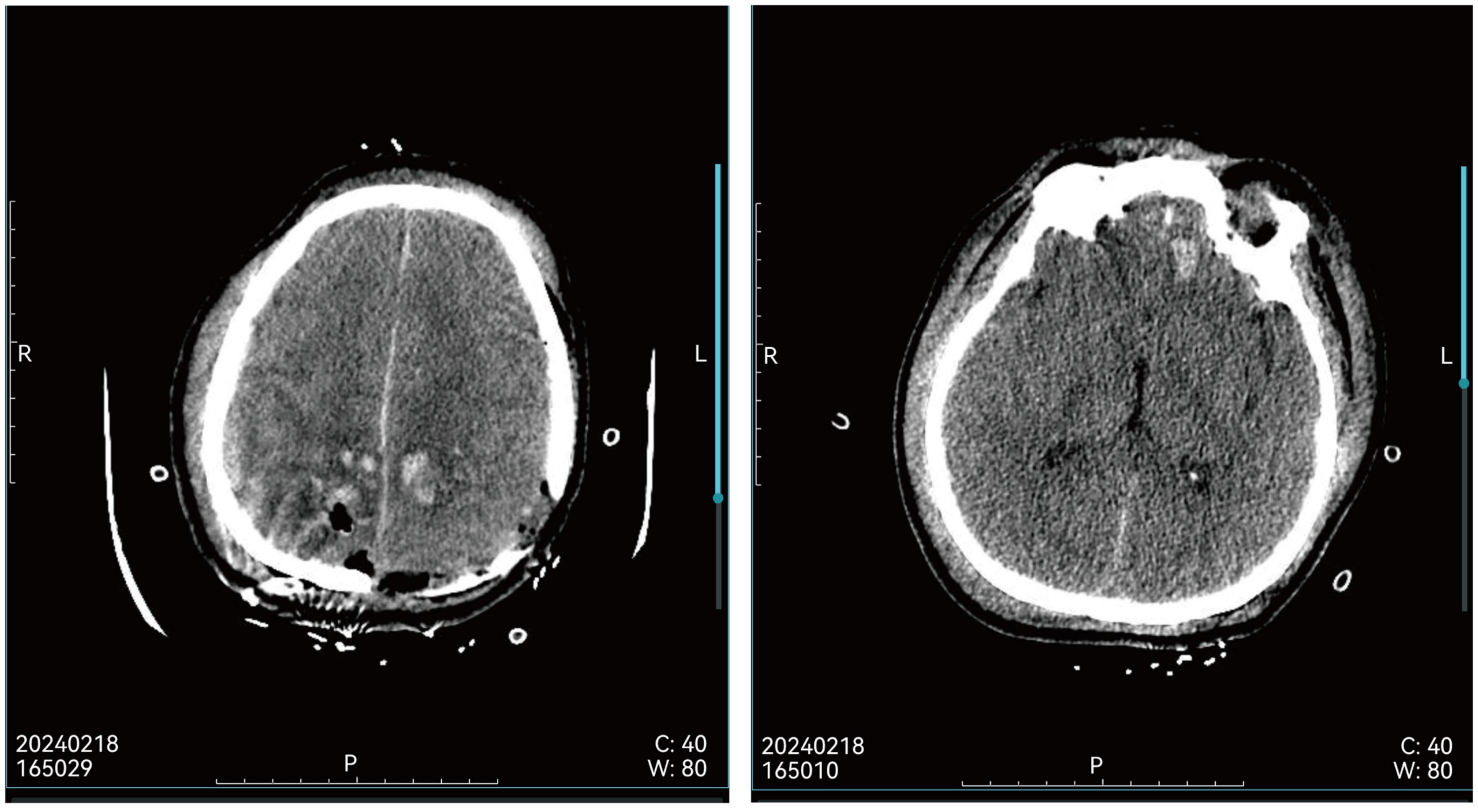
Head CT showed multiple cerebral injuries in the bilateral parietal lobes, left frontal lobe, and left temporal lobe after trauma.

A 58-year-old male farmer with no significant past medical history sustained severe traumatic brain injury (TBI) from a 4-m fall during work at home on February 18, 2024. Emergency cranial CT scan (Figure 1) 3 h post-injury revealed multiple cerebral contusions in bilateral frontoparietal lobes and left temporal lobe, left parietotemporal subdural/extradural hematoma, traumatic subarachnoid hemorrhage, and skull fractures. Neurological physical examination showed disturbance of consciousness (GCS score of 7), unresponsiveness to verbal stimuli, abnormal mental behavior (crying and agitation), no response to pain, preserved physiological reflexes, bilateral pupils equal in size (3 mm diameter) with sluggish light reflex, limb weakness, and multiple episodes of non-projectile vomiting. The patient underwent emergent neurosurgical interventions including decompressive craniectomy, evacuation of multiple left intracerebral hematomas, dural repair, and venous sinus repair. After 6 days of intensive care unit (ICU) treatment (including anti-infection, neuroprotection, dehydration, fluid resuscitation, homeostasis maintenance, and nutritional support), the patient was transferred to the general neurosurgery ward for 1 month of symptomatic neurorehabilitation.

Upon transfer to the rehabilitation unit at a month post-injury, neurological assessment indicated clear consciousness and normal cranial nerve examination. Motor examination revealed Medical Research Council (MRC) scale grades: right upper limb 0, left upper limb 2, and bilateral lower limbs 0. Generalized hypotonia was observed with complete dependence in activities of daily living (ADL score 0). Physiological reflexes (biceps, triceps, brachioradialis, and patellar tendons) were normoactive without pathological reflexes. Follow-up imaging confirmed residual changes from cerebral contusions. After 4 months of standardized rehabilitation (July), motor function showed partial improvement: bilateral lower limbs achieved limited flexion range; MRC grades improved to right upper limb 1, right lower limb 2+, left lower limb 2. However, mild hypertonia emerged in the lower extremities.

At 8 months post-injury (October), the patient was readmitted with progressive hypertonia. Clinical features included persistent bilateral lower limb rigidity [Modified Ashworth Scale (MAS) grade 3], frequent nocturnal spasms (5–7 episodes/night), impaired assisted standing, and sleep disruption severely affecting quality of life. Pharmacotherapy (eperisone 150 mg/day, oxcarbazepine 150 mg bid) proved ineffective. Neurological physical examination documented MRC grades: right lower limb 2+, right upper limb 1, left lower limb 2, left upper limb 4; bilateral patellar hyperreflexia without pathological reflexes. ADL indicated severe dependence (score 15). Electromyography suggested impaired sensory pathways in the lower limbs. Tone assessment showed mild right upper limb hypotonia and significant bilateral lower limb hypertonia (MAS 3). Physiological reflexes remained normoactive without pathological signs.

A trial epidural spinal cord stimulation electrode was implanted, demonstrating effective tone reduction. Due to financial considerations, the test electrode was removed on October 10, 2024, and muscle tone promptly deteriorated to complete stiffness. This clinical course illustrates the characteristic progression of post-TBI spasticity: initial flaccid paralysis, intermediate tone recovery, and late-stage refractory hypertonia. Despite 9 months of guideline-directed pharmacotherapy and rehabilitation, spasticity progressed to a pharmacoresistant state (MAS ≥ 2 with functional impairment), meeting criteria for neuromodulation intervention. Following informed consent, permanent lumbar SCS implantation was indicated.

### Ethical statement

The SCS implantation procedure was conducted in accordance with the principles of the Declaration of Helsinki and approved by the Ethics Committee of Yueyang Traditional Chinese Medicine Hospital (YZYEC[2024]P0039).

### Surgical procedure and stimulation protocol

**Figure 2:**
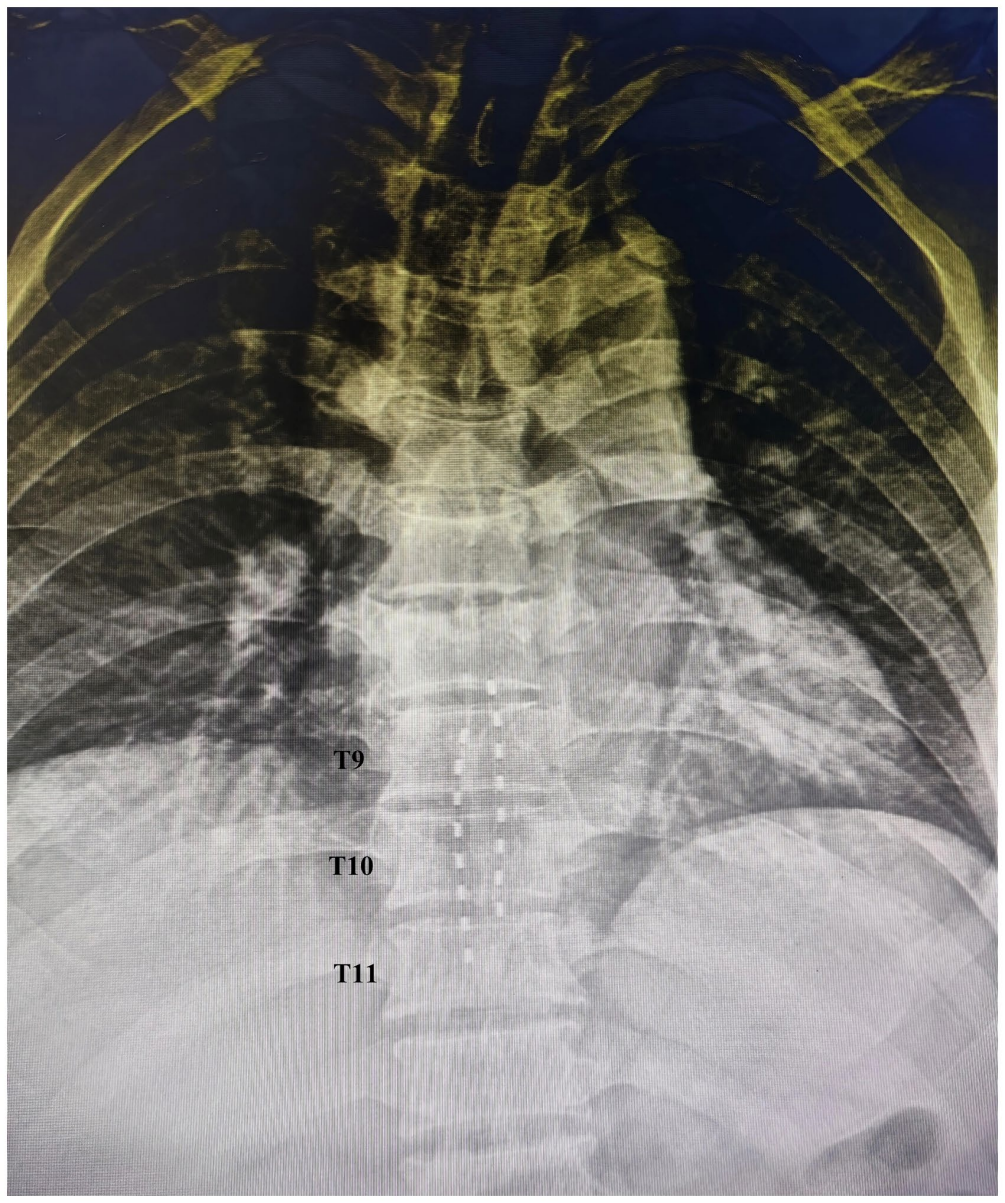
X-ray image of two eight-contact percutaneous leads placed in the T9–T11 level.

Preoperative MRI of the head and cervicothoracic spine showed abnormal signal lesions in bilateral frontoparietal junctions, left temporal lobe, and bilateral frontal gyri recti, consistent with traumatic history, and compression fractures at T6, T7, T10, and T12. Surgery was performed under local anesthesia and two percutaneous epidural electrodes (PINS, G122R, China) was implanted at T9–T11 (Figure 2), as the lumbar enlargement spans T9–L1 vertebral levels, and T9–T11 electrodes cover most lower limb neuronal pools.

**Figure 3:**
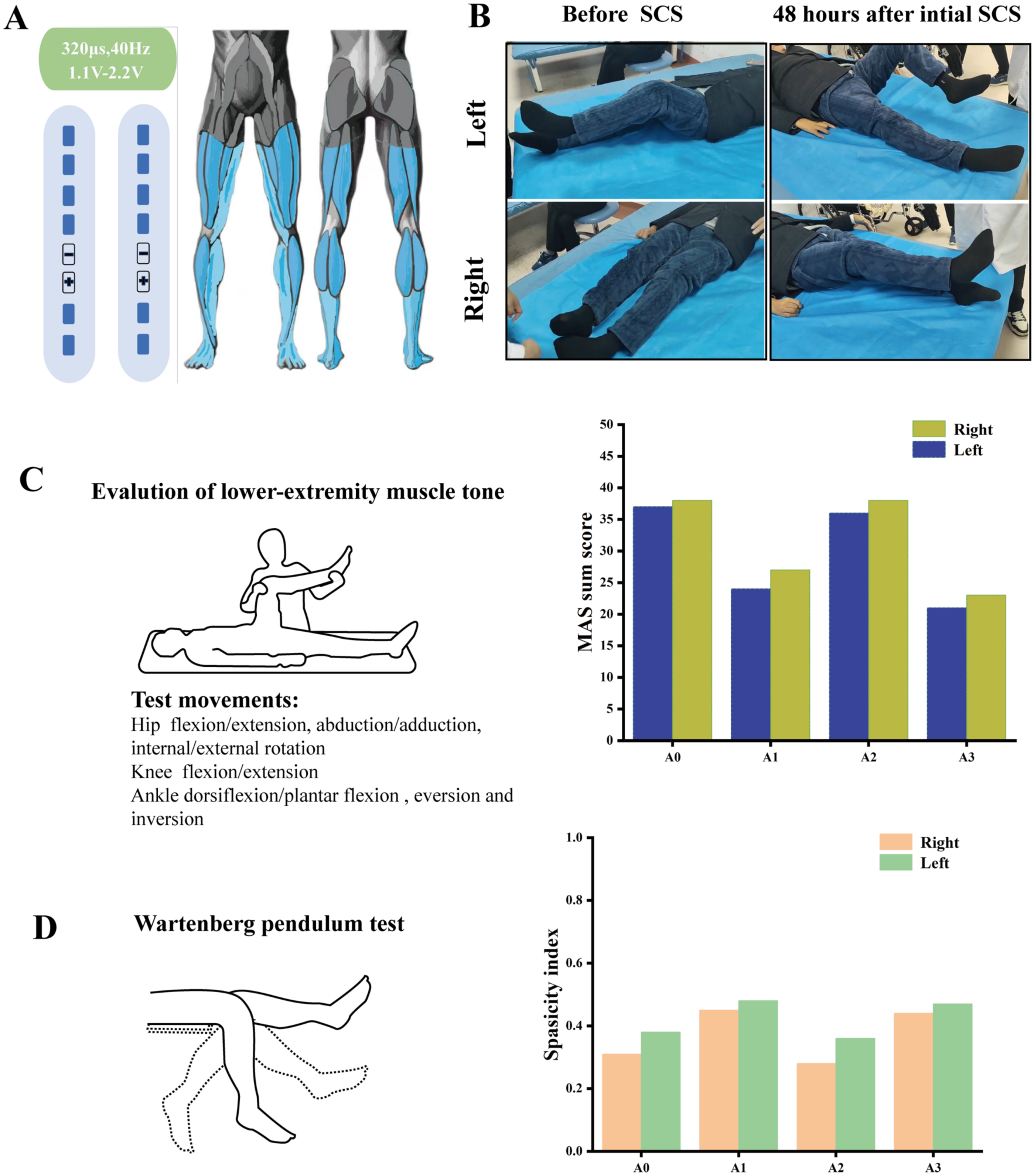
Evaluation of spasticity following the initial 48-h spinal cord stimulation (SCS), conducted at different time points: before electrical stimulation (A0), 48 h after stimulation initiation (A1), 30 min after stimulation cessation (A2), and 2 h after resumption of stimulation (A3). **(A)** The designed SSC neuromodulation parameters, under which the distribution range of numbness in the patient’s lower limbs was shown in the schematic diagram. **(B)** Improvements in the patient’s range of motion (ROM) after 48 h of treatment. **(C)** Total Modified Ashworth Scale (MAS) scores for passive movements of muscles across 12 motions involving the hip, knee, and ankle joints. **(D)** Spasticity index (a value ≥1 indicates a non-spastic state, while a value approaching 0 signifies severe spasticity) derived from the Wartenberg pendulum test, which detects tone in muscle groups across the knee joint.

Immediate postoperative programming tested electrode configurations (cathode/anode arrays) for muscle recruitment and selectivity. The patient lay supine while single stimuli (pulse width 320 μs, frequency 2 Hz, minimum system frequency) were applied, with amplitude increased from below sensory threshold to motor response saturation or discomfort (0.1 mA, 10 pulses/step). Optimal configurations were determined to enhance active monoarticular and multiarticular movements. Initially set at 20 Hz, frequency was increased to 100 Hz in 10 Hz steps, with 40 Hz finalized based on patient feedback. As shown in Figure 3A, the final protocol induced bilateral lower limb paresthesia coverage and maximized joint range of motion.

### Follow-up and data acquisition

The initial programming and testing of electrode configurations commenced approximately 5 h after surgery, once the patient was fully alert and able to provide consistent feedback. Thereafter, continuous, sub-motor-threshold stimulation was delivered without interruption unless the patient requested a pause (e.g., for sleep or personal comfort). The amplitude was set at a level that elicited comfortable paresthesia without inducing muscle contraction. Fine-tuning of the electrode configurations (anode/cathode selection) and amplitude was performed periodically based on the patient’s subjective report of paresthesia coverage and comfort.

The first assessment was conducted 48 h after the initiation of electrical stimulation. The efficacy was evaluated 48 h after the initial electrical stimulation with a stimulator on/off test protocol at four time points: Before the electrical stimulation (A0), 48 h after stimulation (A1), 30 min after stimulation shutdown (A2), and 2 h after restimulation (A3).

*Muscle tone*: The lower limb muscle tone of patients was quantitatively evaluated using the Modified Ashworth Scale (MAS). MAS quantified lower limb tone for 12 independent movements (hip flexion/extone, abduction/adduction, internal/external rotation; knee flexion/extone; ankle dorsiflexion/plantar flexion, eversion and inversion).

Muscle strength was evaluated using the Medical Research Council (MRC) scale, with assessments performed bilaterally on the following leg muscles: rectus femoris (RF), gluteus maximus (GLmax), gluteus medius (GLmed), biceps femoris (BF), semimembranosus (SM), vastus lateralis (VL), lateral gastrocnemius (LA), gastrocnemius medialis (GM), and tibialis anterior (TA).

*Wartenberg pendulum test*: The patient sat, trunk flexed 30°, legs released from horizontal to swing passively. Spasticity index was calculated from three trials/limb (10-s intervals). The spasticity index was derived from three key knee angles measured during the Wartenberg pendulum test: the initial horizontal position (Start), the first reversal point from flexion to extension (Flex), and the final resting angle (Rest).

$$\text{spasticity index}=\frac{F\mathrm{lex}-\text{start}}{1.6*(\text{Rest}-\text{start})}$$

*Spasm frequency*: The frequency of spasm episodes in patients was quantitatively evaluated daily using the Penn Spasm Frequency Scale (PSFS) to comprehensively monitor the dynamic changes of spasm symptoms.

Post-discharge weekly home follow-ups continued standardized assessments to ensure data continuity. All outcome assessments—including the Modified Ashworth Scale (MAS), Medical Research Council (MRC) scale, Penn Spasm Frequency Scale (PSFS), and Wartenberg pendulum test—were performed according to their standardized published guidelines. To ensure consistency, every evaluation was conducted by the same board-certified physiatrist with extensive experience in neurorehabilitation and spasticity management.

## Results

The neuromodulation was initiated after determining optimal electrical stimulation parameters. Therapeutic effects emerged after 48 h stimulation, primarily characterized by reduced lower limb muscle tone. As shown in Figure 3B, the range of motion (ROM) of the hip and knee joints in the patient’s lower extremities was significantly increased compared with that before SCS, with the ROM of the left lower extremity being better than that of the right. Unfortunately, the patient exhibited severe ankle varus, and the improvement in ankle ROM was not significant after the initial 48-h SCS. Following the initial 48-h stimulation, detailed physical examinations were conducted at four time points: before the first stimulation (A0), 48 h after initial stimulation (A1), 30 min after stimulation shutdown (A2), and 2 h after restimulation (A3). Assessments included muscle tone, Wartenberg pendulum test. As shown in Figures 3C,D, after the first 48-h lumbar enlargement spinal cord stimulation, the muscle tone of 12 joint movements across 3 joints of the patients’ lower limbs were evaluated using the Modified Ashworth Scale (MAS) scores. The total MAS scores of muscle tone in the left and right lower limbs of the patients decreased from 38 and 37 points to 19 and 20 points, respectively. Spasticity index, reflecting calf swing dynamics, increased from 0.31 and 0.38 at A0 to 0.45 and 0.48 at A1. Notably, 30 min after stimulation cessation, lower limb muscle tone rebounded, with MAS scores rising to 37, and spasticity index dropping to 0.28 and 0.36. Two hours after restimulation, therapeutic effects reappeared, with MAS scores decreasing to 20 and 20, and spasticity index recovering to 0.44 and 0.47. Additionally, the patient indicated that the duration of sleep onset at night increased from 2 h to approximately 3 h on the first postoperative day. There were no notable alterations observed in the muscle strength of the patient’s lower extremities, nor in the muscle strength and tone of the upper extremities.

**Figure 4:**
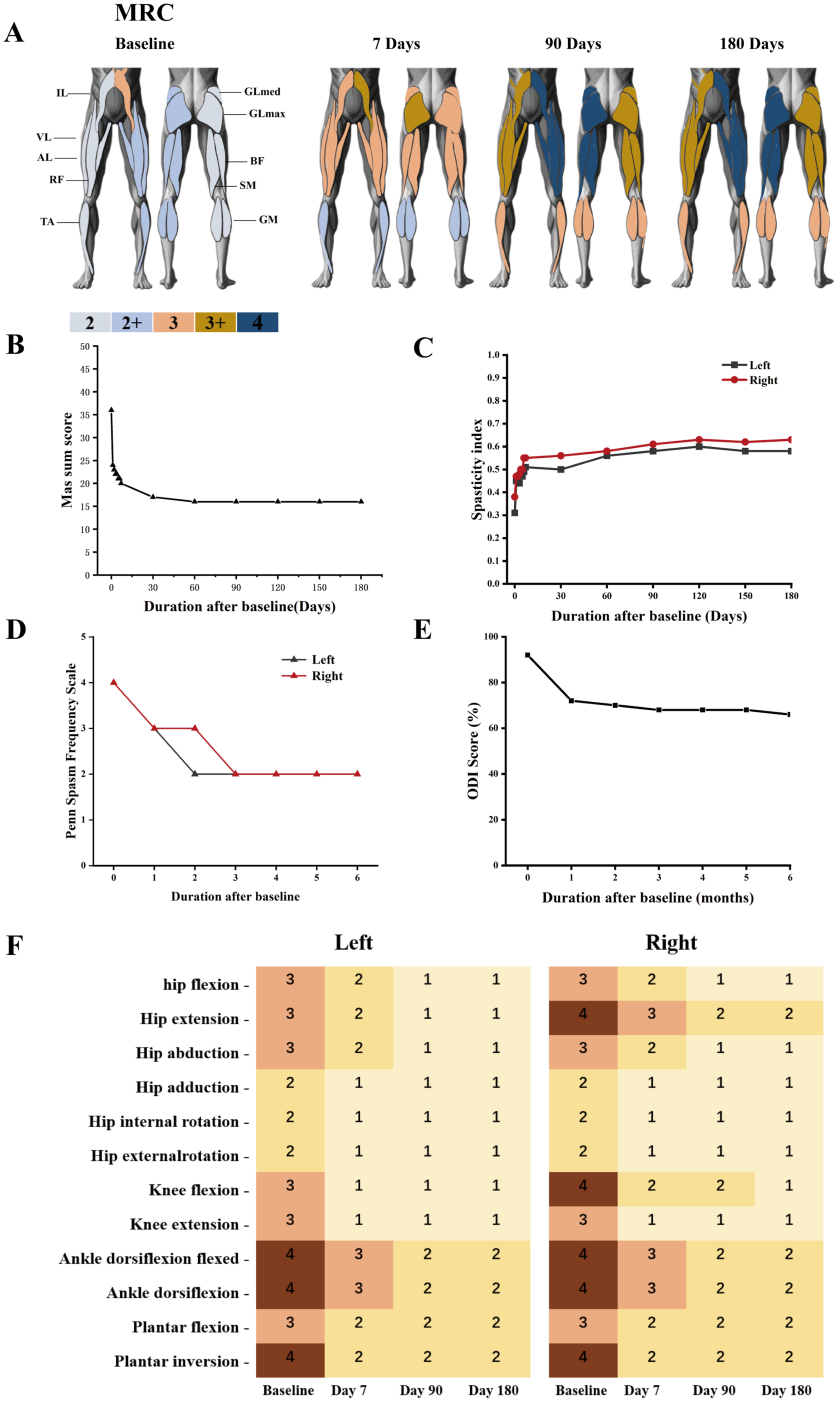
The effect of 6-month lumbosacral enlargement spinal cord stimulation (SCS) combined with home-based rehabilitation therapy in the patient. **(A)** MRC scores of the patient’s leg muscles during the 6-months rehabilitation program, all measured without stimulation. Each assessment refers to the number of days after epidural electrode implantation. The first assessment represents the baseline evaluation before the epidural electrode implantation. Different colors represent different MRC scores. **(B)** Total modified Ashworth Scale (MAS) score, **(C)** spasticity index, **(D)** Penn Spasm Frequency Scale, and **(E)** Oswestry Disability Index (ODI) during the follow-up period. **(F)** Changes in modified Ashworth Scale (MAS) scores for different movements of the hip, knee, and ankle joints.

**Figure 5:**
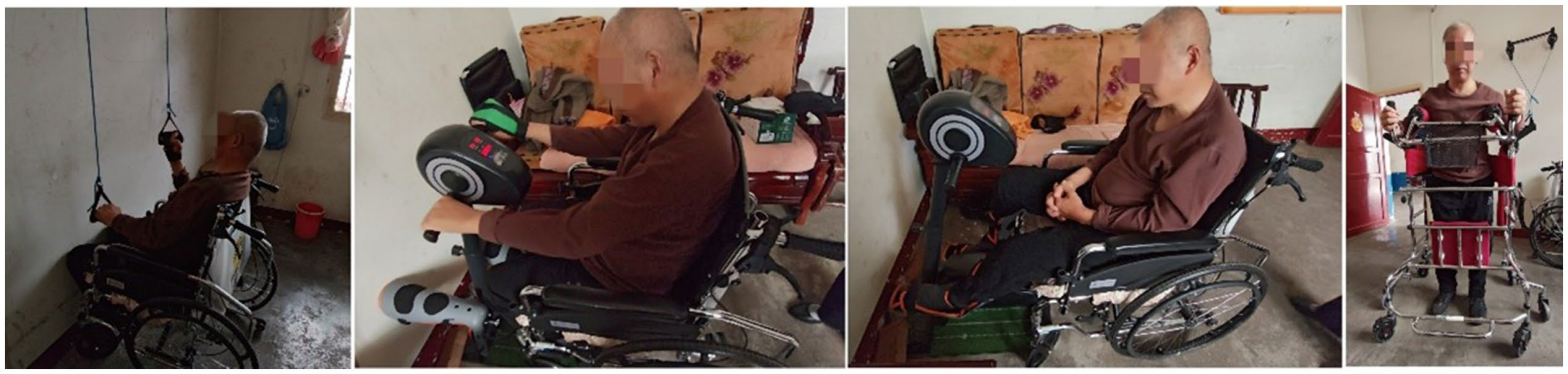
Photographs of the patient’s home-based rehabilitation equipment training, with each training program lasting for half an hour every day.

Consistent follow-ups were performed daily during the first postoperative week and monthly after discharge. The changes in muscle strength of the patient’s lower extremities are shown in Figure 4A. With the progression of treatment, the muscle strength of the patient’s lower extremities showed an increase. As shown in Figure 4B, total MAS scores for lower limb muscle tone declined rapidly within the first month of stimulation and stabilized thereafter. At 6 months postoperatively, the total MAS scores for the right and left legs were 16 and 15, respectively. The changes in MAS scores for each different movements of the patient’s lower limbs were shown in Figure 4F. Overall, as the treatment time increases, the MAS scores show a downward trend. The patient’s Oswestry Disability Index (ODI) improved from 85% (severe disability at admission) to 60% after 6 months of treatment, maintaining stability thereafter (Figure 4C). The spasticity index of bilateral lower limb increased significantly during the first week of stimulation and then stabilized at 0.44 and 0.47 (Figure 4D). Patient-reported spasm frequency, evaluated by the Penn Spasm Frequency Scale, decreased from 4 points preoperatively to a stable 2 points (Figure 4E). In contrast to preoperative insomnia due to spasticity, the final follow-up revealed the patient could sleep continuously for over 6 h at night. Improved spasticity enabled the patient to perform home-based instrumental rehabilitation, including 30-min daily sessions of upper limb lat pulldowns, bilateral lower limb cycling, upper limb cycling, and assisted standing (Figure 5). Following the reduction in spasticity, the patient was able to perform home-based instrumental rehabilitation. The ability to engage in these activities correlated with significant functional improvements.

## Discussion

In addition to TBI, various other central nervous system diseases such as spinal cord injury, stroke, multiple sclerosis (MS), cerebral palsy, amyotrophic lateral sclerosis (ALS), hypoxic encephalopathy, and phenylketonuria are also frequently accompanied by spasticity. Although multiple studies indicate that spinal cord stimulation (SCS) provides therapeutic benefits for spasticity patients with over 25 neurodegenerative or traumatic etiologies, its clinical application remains controversial due to the low quality of existing evidence (Hofstoetter et al., 2021; Lin et al., 2022).

SCS was initially used for pain management, with attempts to apply it in spasticity treatment emerging in the 1970s and 1980s (Knotkova et al., 2021). Subsequent advances in intrathecal baclofen drug delivery (ITB) therapy led to a gradual decline in clinical attention toward SCS. However, SCS offers notable advantages over ITB therapy. On one hand, pharmacotherapeutic approaches are associated with issues such as side effects and patient intolerance; on the other hand, the difficulty of drug dose titration is significantly greater than the calibration of device parameters. Furthermore, emerging research evidence in recent years has revealed the regulatory effect of SCS on motor neural circuits, prompting the academic community to re-evaluate its application potential in the field of spasticity treatment (Mayorova et al., 2023).

The current state of the art in SCS for spasticity management is characterized by promising yet preliminary evidence, with a notable resurgence of research interest. Recent studies have moved beyond early empirical approaches to explore mechanism-informed protocols, such as high-frequency or dorsal root ganglion stimulation, primarily in spinal cord injury or stroke, showing potential in reducing hypertonia and improving voluntary motor control (Zhang et al., 2024; Greiner et al., 2021; Gazzeri et al., 2024). However, the field is still constrained by a lack of high-level evidence. The literature predominantly consists of small case series, heterogeneous patient populations, and variable outcome measures, making robust meta-analysis difficult. Key knowledge gaps persist regarding optimal patient selection, stimulation paradigms (e.g., frequency, amplitude, target), long-term efficacy, and cost-effectiveness. Consequently, SCS is not yet considered a first-line standard therapy for refractory spasticity in most guidelines, and its application often remains within specialized centers or research protocols. Crucially, evidence specifically focusing on TBI-induced spasticity is particularly scarce.

Spasticity encompasses intricate neurobiological processes. While notable progress has been made in current understanding of this condition, a comprehensive elucidation of its underlying mechanisms remains challenging. The pathophysiological mechanisms underlying spasticity can be categorized into two types: spinal mechanisms involving motor neurons and interneurons, and supraspinal and suprasegmental mechanisms. Essentially, it results from the combined effects of the loss of descending inhibitory control and the reorganization of spinal neural circuits following central nervous system injury.

The precise mechanisms underlying the therapeutic effects of SCS in spasticity remain incompletely elucidated, and a unified theoretical framework is still lacking. Building on its research foundation in pain management and findings from select studies on SCS for spasticity, we have put forward several potential mechanisms of action.

SCS activates primary afferent fibers within the dorsal columns (DC), subsequently exciting inhibitory interneurons (predominantly GABAergic neurons) in the spinal dorsal horn. This process enhances presynaptic inhibition: GABA release suppresses calcium influx at Ia afferent terminals, reducing glutamate exocytosis and thereby diminishing excitatory input to motor neurons. Concurrently, SCS may restore reciprocal Ia inhibition by facilitating inhibitory interneuron-mediated suppression of antagonist MNs and alleviating pathological co-contraction. Recent studies have substantiated the modulatory influence of SCS on both presynaptic and postsynaptic inhibitory pathways. In chronic spasticity, MNs experience sustained depolarization as a result of alterations in ion channel structure and function. SCS potentially counteracts this by augmenting potassium efflux or modulating calcium channels, thereby stabilizing MN membrane potentials and suppressing spinal hyperexcitability through reduced plateau potential generation. In addition, SCS may play a role in modulating the balance of descending control pathways. Stimulation of the DC has the potential to activate the brainstem’s inhibitory centers, including the ventromedial reticular formation located in the medulla. This activation may facilitate the reactivation or compensatory enhancement of the descending inhibitory pathway known as the dRST. Studies have demonstrated that SCS can increase the concentrations of 5-HT and NA in cerebrospinal fluid. These monoaminergic neurotransmitters are pivotal to the “gain control system” of motor neurons: 5-HT enhances the persistent inward current (PIC) in MNs, while NA modulates the excitability of interneurons. SCS likely reestablishes spinal excitatory-inhibitory homeostasis by restoring physiological monoamine levels. Notably, studies demonstrate SCS-enhanced functional connectivity between brainstem reticular formations and the contralesional premotor cortex (Brodmann area 6). As this pathway constitutes the core corticoreticular tract responsible for spinal reflex inhibition, its potentiation may correct the imbalance between descending inhibitory and facilitatory signals in spasticity (Mohammed and Hollis, 2018; Braaß et al., 2025; McIntosh et al., 2024). Our case report cannot definitively confirm any single mechanism but provides a valuable clinical observation.

This case report provides the first detailed description of the remarkable efficacy of lumbar enlargement spinal cord stimulation combined with rehabilitation therapy for refractory spasticity following severe TBI. In this combined approach, the patient exhibited a reduction in muscle tone from grade 3 to grade 1+, a 70% decrease in spasm frequency, and simultaneous improvements in motor function (muscle strength and joint range of motion) and ADL capacity. These therapeutic benefits remained stable throughout the 6-month follow-up period. These findings offer novel evidence supporting the application of SCS in spasticity management and provide valuable pioneering experience for its extone to the TBI population. However, the findings of this report should be interpreted within the context of its single-case design. The lack of a control group and the concurrent administration of home-based rehabilitation preclude definitive causal attribution of functional improvements solely to spinal cord stimulation. Furthermore, while outcome assessments followed standardized methods, they were performed by an unblinded evaluator, which may introduce observation bias. These limitations highlight the need for future investigations incorporating larger, controlled cohorts—ideally through multicenter randomized trials—to confirm efficacy, optimize stimulation parameters, and clarify the role of SCS within integrated rehabilitation protocols for post-TBI spasticity.

## Conclusion

This case demonstrates that lumbar enlargement spinal cord stimulation constitutes a safe and effective therapeutic approach for refractory lower limb spasticity following TBI. Beyond being associated with significant reductions in muscle tone and spasm frequency, the application of SCS, in combination with rehabilitation, correlated with improvements in motor function and activities. With ongoing advancements in neuromodulation technologies and the accumulation of clinical evidence, SCS is expected to become an important component of multidisciplinary management of spasticity.

## Data Availability

The raw data supporting the conclusions of this article will be made available by the authors, without undue reservation.
